# Targeting the non-enzymatic function of DPP4 via a nanobody inhibits cerebral cavernous malformation progression

**DOI:** 10.1186/s12951-026-04898-x

**Published:** 2026-08-05

**Authors:** Chunyang Men, Ziyu Xiong, Qianpeng Duan, Jiaxin Cheng, Xiqing Luo, Bao Yang, Ying Li, Cong Yan, Yuwen Wang, Yongqing Ye, Rui Bi, Zifeng Dai, Silengtu Hu, Xia Zhao, Wenzhong Du, Dongdong Zhang, Changbin Shi

**Affiliations:** 1 Department of Neurosurgery, First Affiliated Hospital of Harbin Medical University, 23 Youzheng Street, Nangang District, Harbin, 150001 Heilongjiang province China; 2 NHC Key Laboratory of Cell Transplantation, First Affiliated Hospital of Harbin Medical University, Harbin, Heilongjiang China; 3 Department of Critical Care Medicine, First Affiliated Hospital of Harbin Medical University, Harbin, Heilongjiang China; 4 First Affiliated Hospital of Ningbo University, Zhejiang, China

**Keywords:** Cerebral cavernous malformation, DPP4, Nanobody, Vascular remodeling, Single-cell RNA sequencing, generative AI

## Abstract

Cerebral cavernous malformations (CCMs) cause hemorrhagic stroke and lack effective pharmacological therapies. Although multiple extracellular stimuli contribute to CCM progression, how these signals are integrated across the plasma membrane remains unclear. Through transcriptomic analysis, we identified *dipeptidyl peptidase-4 (DPP4)* as a hub gene that may link extracellular stimuli to endothelial dysfunction, with elevated expression in CCM lesions. DPP4 is a type Ⅱ transmembrane glycoprotein that possesses both enzymatic and non-enzymatic functions. DPP4 knockdown reverses endothelial dysfunction in vitro and suppresses CCM progression in vivo. Single-cell transcriptomic analysis further reveals that DPP4 promotes pro-angiogenic signaling and is associated with caveolin-1 (CAV1). Mechanistically, our data suggest that DPP4 promotes CCM pathogenesis via a non-enzymatic interaction with CAV1, largely independent of its canonical enzymatic activity. This interaction promotes CAV1 phosphorylation and downstream FAK signaling activation. To selectively target the predicted interface epitope on DPP4, we developed a structure-guided nanobody (DC499-mFc) and functionally disrupted the DPP4–CAV1 interaction. This nanobody-based strategy effectively attenuates CCM progression in vivo. Collectively, these findings suggest that DPP4 contributes to CCM progression and point to nanobody‑based disruption of the non‑enzymatic DPP4–CAV1 interaction as a potential therapeutic strategy for CCM.

**Graphical Abstract:**

**Figure:**
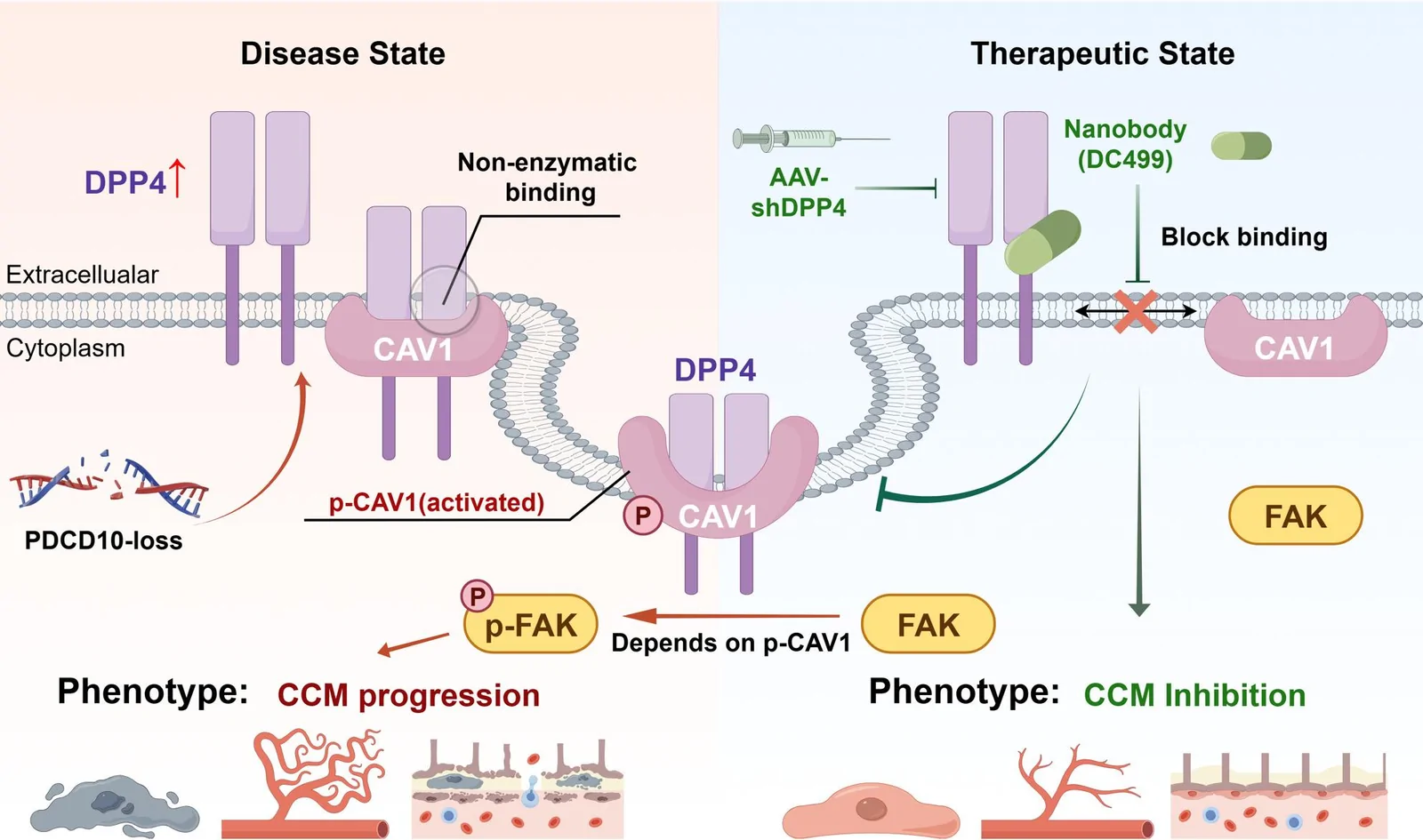

**Supplementary Information:**

The online version contains supplementary material available at https://doi.org/10.1186/s12951-026-04898-x.

## Introduction

Cerebral cavernous malformations (CCMs) are common cerebrovascular anomalies affecting approximately 0.5% of the general population [1, 2]. Characterized by clusters of excessively dilated, thin-walled, and structurally abnormal capillaries within the central nervous system [3], CCMs predispose patients to a lifetime risk of recurrent spontaneous hemorrhages, seizures, and progressive focal neurological deficits [4]. The disease is primarily driven by loss-of-function mutations in one of three CCM genes (*KRIT1/CCM1*,* Malcavernin/CCM2*,* or PDCD10/CCM3*), and classified into two forms: familial CCM caused by germline mutations and sporadic CCM caused by somatic mutations [1, 3]. Currently, surgical resection is the standard first-line treatment option for accessible lesions. However, it is often unsafe or infeasible for deep or multifocal CCM lesions, highlighting an urgent need to understand disease mechanisms and develop effective pharmacological therapies.

The pathogenesis of CCM is driven by endothelial dysfunction, which is characterized by compromised barrier integrity, aberrant cell migration, and pathological angiogenesis [3, 5]. The mouse model with brain EC-specific deletion of *Pdcd10* faithfully reproduce the pathological features of the human disease [6]. Recent studies have established that dynamic stimuli from the extracellular environment contribute to endothelial dysfunction in CCM. These stimuli include mechanical stress, local hypoxia, neuroinflammation, and pro-angiogenic signals such as VEGFA and ANGPT2 [7–11]. Despite these advances, the key transmembrane signaling mechanisms by which ECs integrate extracellular stimuli and initiate pathological cascades remain unclear. We hypothesize that specific signaling molecules enriched on the CCM endothelial cell membrane are upregulated in the pathological microenvironment and function as critical signaling regulators, enabling ECs to sense extracellular stimuli and transition from a quiescent to an aberrant migratory state. Identifying such regulators is essential for the discovery of novel, druggable therapeutic targets.

To this end, we conducted transcriptomic analyses of human CCM lesions and identified *dipeptidyl peptidase 4* (*DPP4*, also known as *CD26*) as a potential node linking extracellular stimulus responses to endothelial migration. DPP4, a pleiotropic type II transmembrane glycoprotein, is known for its enzymatic role in insulin resistance by cleaving GLP-1 [12]. Emerging evidence highlights its critical non-enzymatic functions in signal transduction [13, 14]. Moreover, inhibition of DPP4 enzymatic activity alone is insufficient to replicate the effects of direct DPP4 knockdown on downstream signaling, suggesting that additional mechanisms are involved [13, 15]. The precise molecular basis of its non-enzymatic signaling functions therefore remains to be elucidated. DPP4 has been reported to interact with Caveolin-1 (CAV1) [16–18], a principal scaffolding protein of membrane caveolae involved in signal transduction and angiogenesis [18, 19]. Given the critical role of CAV1 in mediating extracellular signaling, we hypothesized that the DPP4–CAV1 axis acts as a key transmembrane signaling integrator driving the endothelial dysfunction in CCM lesions.

In this study, we demonstrate that DPP4 is significantly upregulated in the CCM-lesional ECs, and uncover a previously unrecognized mechanism: DPP4 promotes CCM pathogenesis primarily through a non-enzymatic interaction with CAV1 on the endothelial membrane, largely independent of its well-known enzymatic activity. This interaction is associated with phosphorylation of CAV1 at the Tyr14 site, which may promote the transmission of pro-angiogenic signaling and trigger the activation of focal adhesion kinase (FAK), ultimately leading to abnormal endothelial migration and barrier disruption. Notably, pharmacological inhibition of DPP4’s enzymatic activity failed to halt disease progression, underscoring the need to specifically disrupt the DPP4–CAV1 interface. To achieve this, we utilized RFdiffusion [20] (a generative AI tool) to develop an engineered nanobody (DC499-mFc) specifically targeting the predicted non-enzymatic CAV1-binding region of DPP4. By blocking the interaction between DPP4 and CAV1, DC499-mFc significantly reduced the burden of lesions and bleeding in vivo. In summary, our study reveals a novel non-enzymatic transmembrane signaling pathway in the pathophysiology of CCM, offering a promising and specific therapeutic strategy for this intractable cerebrovascular disease.

## Methods

### Bioinformatics analysis

RNA-seq datasets (GSE137596, GSE137425, and GSE204979) were obtained from the Gene Expression Omnibus (GEO) database. GSE137596 (10 CCM and 4 control samples) was used as the discovery cohort, while GSE137425 and GSE204979 served for independent validation. Gene sets were retrieved from MSigDB, with a focus on *GOBP_ENDOTHELIAL_CELL_MIGRATION* (GO:0043542) and *GOBP_REGULATION_OF_RESPONSE_TO_ EXTERNAL_STIMULUS* (GO:0032101). Differential expression gene (DEG) analysis was performed using the “limma” R package, and genes with |log2FC| > 2 and FDR < 0.05 were considered differentially expressed. Protein–protein interaction networks were constructed using STRING database and visualized in Cytoscape (v3.10.3).

### Human specimens

The specimens were obtained from the First Affiliated Hospital of Harbin Medical University, comprising 5 CCM lesions and 3 non-lesional control brain samples. The Declaration of Helsinki and its amendments were followed. The study was approved by the Institutional Review Board of the First Affiliated Hospital of Harbin Medical University (No. 2025331). All participants gave written consent for their surgical specimens to be used for research purposes.

### Genetically modified animals

Brain endothelial-specific *Pdcd10* conditional knockout mice (*Pdcd10*^*BECKO*^) were generated to develop CCM lesions in vivo model by crossing mice expressing tamoxifen-inducible Cre recombinase under the control of the *Slco1c1* promoter (*Slco1c1*-CreERT2) with mice harboring a loxP-flanked *Pdcd10* allele (*Pdcd10*^*fl/fl*^) [6]. All mouse strains were based on *C57BL/6J*. CCM models were induced by intragastric administration of 25 µg 4-hydroxytamoxifen (H7904, Sigma-Aldrich) at postnatal day 1 (acute model) or day 3 (chronic model). *Pdcd10*^*fl/fl*^ and *Pdcd10*^*BECKO*^ mice were generated by Mendelian inheritance, and they were randomly assigned for subsequent experiments at P6. Mice were euthanized at P28 under tribromoethanol anesthesia. All animal procedures were approved by the Institutional Animal Care and Use Committee (IACUC) of the First Affiliated Hospital of Harbin Medical University (No. 2024068) and conducted in accordance with the NIH guidelines. Animals were housed under standard conditions (12 h light/dark cycle, 20–24 °C, 45–65% humidity) with free access to food and water.

### Cell culture

Human cerebral microvascular endothelial cells hCMEC/D3 were cultured in endothelial cell medium (ScienCell, #1001). HEK293T cells were cultured in DMEM (Gibco) supplemented with 10% FBS. All the cells were cultured at 37 °C in a humidified atmosphere with 5% CO₂.

### Lentivirus infection

shRNA targeting *PDCD10* and control sequences were cloned into VP013-U6-MCS-CMV-ZsGreen-PGK-PURO vectors. The packaged lentivirus was provided by General Biol Co., Ltd. hCMEC/D3 cells were infected at ~ 30% confluence following the manufacturer’s protocol.

### siRNA and plasmid transfection

siDPP4 or control siRNA was transfected using Lipofectamine 3000 (Thermo, #L3000001). Cells at ~ 50% confluence were incubated with transfection mixtures for 6 h, followed by medium replacement; assays were conducted at 72 h. The plasmids were constructed in pcDNA3.1 vector, including CAV1(wt)-Flag, CAV1(Y14F)-Flag, DPP4(wt)-HA, DPP4(S630A)-HA. These plasmids were transfected at ~ 90% confluence using Lipofectamine 3000. Experiments were performed after 48 h.

### AAV treatment

The adeno-associated virus (AAV) vector used was AAV-BI30 obtained from Vigenebio (Wuxi, China). Mice received 1 × 10¹¹ vg AAV-shDPP4 or AVV-vector via orbital vein injection at P6 to assess the effect of DPP4 knockdown on the acute CCM model. 1 × 10¹¹ vg AAV-OE-DPP4 (S624A) or AAV-vector were administrated in the chronic CCM model via orbital vein injection at P6 to assess the role of the non-enzymatic function of DPP4. All animals were harvested at P28.

### scRNA-seq analysis

Cerebellar tissues were collected after perfusion at P28. Single-cell suspensions were prepared using the MobiCube^®^ Single-Cell 3′ Transcriptome Kit. CD31^+^CD45^−^ ECs and CD45^+^ immune cells were sorted by flow cytometry. Library construction and sequencing were performed on MobiNova@-100 platform, followed by initial processing with MobiVision^®^. Sequencing and bioinformatics analyses were conducted by LC-Bio Technologies (Hangzhou) Co., Ltd.

After quality control, 45,338 cells were analyzed using Seurat (v4.1.1). UMAP was applied for dimensionality reduction based on 20 principal components, with resolutions of 1.2 (all cells) and 0.8 (ECs). Marker genes were identified using FindAllMarkers function. Differential expression was assessed using FindMarkers (*p* < 0.01, |log2FC| ≥ 0.26). DEGs were subjected to GO, KEGG, and GSEA analysis. For pseudo-time analysis, trajectory inference was performed using Monocle3, with arteries as the root state. CellChat (v2.1.2) was used to infer cell-cell communication.

### PP2 and sitagliptin treatment

For in vivo treatment, PP2 (MCE, #HY-13805) and sitagliptin (MCE, #HY-13749) were dissolved in DMSO. Either PP2 (1 mg/kg) or sitagliptin (100 mg/kg) was injected intraperitoneally daily in the acute CCM model from P15 to P27, and vehicle served as control. For in vitro treatment, hCMEC/D3 cells were treated with either 2 µM PP2 or 100 nM sitagliptin, and DMSO served as control upon reaching 90% confluence. All experiments were conducted 24 h post-treatment.

### Pathological staining

Perform routine paraffin embedding and sectioning of mouse brain tissue. H&E (Solarbio, #G1120) or Prussian (Solarbio, #G1422) blue staining was performed on the sections according to the manufacturers’ protocols. Images were captured using a VS200 slide scanner (Olympus). For lesion and hemorrhage quantification, ≥ 3 sections per mouse spaced > 100 μm apart were analyzed.

### Immunofluorescence (IF) and immunohistochemical (IHC) staining

Antigen retrieval was performed according to the antibody instructions. For IF, sections were blocked for 1 h with 10% donkey serum (Jackso, #017-000-121) and 0.3% Triton (Solarbio, #T8200) X-100, incubated with primary antibodies overnight at 4℃. After washing, incubate the sections for 1 h with fluorescent secondary antibodies. Mount sections using a mounting medium containing DAPI (Beyotime, #P0131). Images were acquired using an Observer.A1 microscope (Carl Zeiss, Germany) under consistent imaging parameters. For IHC, Staining was performed using a rabbit two-step IHC detection kit (ZSGB-Bio, PV-9001) following the manufacturer’s instructions. Immunostaining was performed on ≥ 3 sections per mouse. Images were captured using a VS-200 slide scanner (Olympus).

### Immunocytochemistry (ICC)

hCMEC/D3 cells were seeded on type I rat tail collagen (Solarbio, #C8062) coated coverslips, fixed with 4% PFA for 15 min, permeabilized with 0.3% Triton X-100 for 15 min, and blocked with 10% donkey serum for 1 h. Cells were incubated with primary antibodies overnight at 4 °C. After washing, they were incubated for 1 h with fluorescent secondary antibodies. Mount coverslips using a mounting medium containing DAPI (Beyotime, #P0131). Images were captured using an Observer.A1 microscope (Carl Zeiss, Germany) or LSM710 (Carl Zeiss, Germany) under identical settings.

### Spheroid sprouting assay

First, prepare ECM medium containing 1.2% methylcellulose (MEC, #HY-125861E). Then mix it with ECM medium containing 5% FBS at a ratio of 1:4 to prepare the spheroid culture medium. Cells were resuspended in the spheroid culture medium and seeded as hanging drops (800 cells/40ul/ droplet) to form spheroids. Prepare the three-dimensional collagen matrices: add 180 µl type I rat tail collagen, 300 µl ECM containing 1.2% methylcellulose, and 120 µl FBS in sequence. After 24 h, spheroids were embedded and cultured in collagen matrices. The images were captured after 12 h using an Observer A1 microscope (Carl Zeiss, Germany).

### Tube formation assay

Coat 24-well plate with basement membrane matrix (ABW, #082704) and solidified at 37 °C. Seed each well uniformly with 2 × 10^5^ hCMEC/D3 cells and culture in ECM medium containing 1% FBS for 12 h. Tube formation images were captured using the EVOS M5000 microscope (Invitrogen). AngioTool was used to analyze tube length.

### Wound healing assay

Cells were seeded into collagen coated 6-well plates. Scratch was performed at ~ 95% confluence, and images were immediately captured. The medium was then replaced with ECM medium without FBS. The images were acquired after 8 h using an Observer.A1 microscope (Carl Zeiss, Germany).

### Western blot

Cells were lysed using 2×Laemmli buffer (Bio-Rad, #1610737) containing protease inhibitor (Selleck, #B14001), phosphatase inhibitor (Selleck, #B15001), and β-Mercaptoethanol. Proteins were separated by SDS-PAGE and transferred to NC membranes. Membranes were blocked with 5% skimmed milk in TBST for 1 h at room temperature, followed by incubation with primary antibodies at 4 °C overnight and HRP-conjugated secondary antibodies. Signals were detected by ECL (Meilunbio, #MA0186).

### Molecular docking and dynamics simulation of DPP4–CAV1 interaction

Crystal structures of DPP4 (4N8D) and CAV1 (7SC0) were obtained from the Protein Data Bank. Protein–protein docking was performed using GRAMM and HDOCK with default parameters [21, 22]. The top two docking models from each method were evaluated using PDBePISA to calculate solvation free energy and buried interface area.

Molecular dynamics (MD) simulations were conducted using GROMACS 2025 with the AMBER99SB-ILDN force field. Energy minimization was performed using the steepest descent algorithm, followed by 1 ns equilibration under constant volume (NVT ensemble) and 1 ns under constant pressure (NPT ensemble). A 100 ns production run was then carried out at 310 K and 1 bar. Structural stability and compactness were assessed by calculating the root mean square deviation (RMSD) and radius of gyration (Rg), respectively. The Gibbs free energy landscape based on RMSD and Rg was further constructed to characterize conformational states.

### Nanobody design and expression

Peptide binders targeting DPP4 were generated as previously described [20]. Briefly, residues on chain B of DPP4 (PDB ID: 4N8D) predicted to mediate interaction with CAV1 were defined as structural hotspots. Guided by these constraints, 300 backbone scaffolds were generated using RFdiffusion, followed by sequence optimization with ProteinMPNN [20]. Structural models of the resulting binders were predicted using AlphaFold3. The binding to the predefined interface of the binders was evaluated by HADDOCK3 [23]. The top five peptide binders were grafted into the CDR3 region of a VHH (nanobody) scaffold. To further optimize binding, random mutations were introduced into the CDR1 and CDR2 regions, generating 500 nanobody candidates [24]. Structures of these candidates were predicted using AlphaFold3, and their interactions with the target interface were modeled by HADDOCK3. Binding affinities were estimated using PRODIGY [25]. The representative nanobody, VHH-DC499, was fused with the Fc domain of mouse IgG to form DC499-mFc, which was then expressed and purified by General Biol Co., Ltd. Briefly, DC499-mFc was expressed in CHO-S cells, and its concentration and yield were determined by measuring absorbance at 280 nm using an extinction coefficient of 1.55 mL·mg^− 1^·cm^− 1^. SDS-PAGE analysis was performed under reducing and non-reducing conditions to determine the molecular weights of the monomeric and dimeric forms, respectively, and to confirm Fc-mediated dimerization. SEC-HPLC was further employed to assess protein purity, retention time, and the oligomeric state of DC499-mFc under native conditions.

### DC499-mFC treatment

For i*n vivo* experiments, DC499-mFc (10 mg/kg) was administered via intraperitoneal injection every other day from P15 to P27 in the acute CCM model, with mFc serving as the control. For in vitro experiments, hCMEC/D3 cells were seeded in 6-well plates and cultured in medium containing either 10 µg/ml VHH-DC499 or control mFc. Cells were harvested for downstream experiment at approximately 90% confluence.

### Pharmacokinetic analysis

C57BL/6 mice (P15) received a single intraperitoneal dose of DC499-mFc (10 mg/kg). Blood was collected at 1, 2, 6, 24, 72, and 120 h post-dose, and plasma concentrations of DC499-mFc were measured using a VHH ELISA Kit (Shangchun Biotechnology, Wuhan, China; #SCB0A015B) following the manufacturer’s standard protocol. Pharmacokinetic parameters, including the maximum observed plasma concentration (C_max_), time to reach maximum concentration (t_max_), area under the concentration–time curve (AUC), terminal elimination half-life (t_1/2_z), total clearance (CLz), and volume of distribution (Vz), were calculated using DAS 2.0 software.

### Co-immunoprecipitation (Co-IP) assays

Cells were lysed in 500 µL of IP lysis buffer (Beyotime, #P0013) per 6 cm dish to prepare protein samples. An aliquot of 50 µL was reserved as the input control, and the remaining lysate was subjected to immunoprecipitation. To assess the interaction between DPP4 and CAV1, HEK293T cells were co-transfected with HA-tagged DPP4 and Flag-tagged CAV1 plasmids. Immunoprecipitation was performed using mouse anti-HA magnetic beads (Beyotime, # P2121) or mouse IgG control beads (Beyotime, # P2171), following the manufacturer’s instructions.

To evaluate the binding of DC499-mFc to DPP4, hCEMC/D3 cell lysates were incubated with Protein A/G magnetic beads (Thermo, # 88802) to precipitate DC499-mFc. The presence of DPP4 in both the precipitate and the supernatant was then analyzed.

### Surface plasmon resonance (SPR) and microscale thermophoresis (MST) assays

SPR assays were performed on a Biacore 8 K instrument (GE Healthcare). Recombinant human DPP4 (MCE, #HY-P72975) was immobilized onto a CM5 sensor chip (Cytiva, #BR-1005-30) via standard amine coupling chemistry. A reference flow cell was subjected to the same activation and blocking procedures in the absence of ligand to enable background subtraction. Serial dilutions of DC499-mFc were prepared in 96-well plates and injected over both flow cells in ascending order (from 0.3125 to 10 nM) at a flow rate of 30 µL/min with an association time of 60 s. After each injection cycle, the chip surface was regenerated with 10 mM glycine-HCl (pH 2.0) for 5 min. Sensorgrams were globally fitted to a 1:1 Langmuir binding model using Biacore Insight Evaluation Software (Cytiva, Marlborough, MA, USA) to derive the association (*k*_*a*_) and dissociation (*k*_*d*_) rate constants and calculate the equilibrium dissociation constant (*K*_*D*_).

MST assays were performed on a NanoTemper Monolith X instrument (NanoTemper Technologies). Recombinant human DPP4 protein was fluorescently labeled using the RED-NHS amine labeling kit (NanoTemper, #MO-L011) according to the manufacturer’s instructions. DC499-mFc was serially diluted (two-fold) to generate 16 concentration gradients, and then mixed with a constant concentration of the labeled DPP4. The mixture was incubated at room temperature for 20 min and then loaded into standard capillaries (NanoTemper, MO-K022). Thermophoretic signals were measured on the Monolith X instrument following the manufacturer’s standard protocol. The dissociation constant (*K*_*D*_) was calculated using the NanoTemper analysis software.

### Statistical analysis

Statistical analyses were performed using GraphPad Prism 9.0.0 software (GraphPad Software, USA). Data are presented as the mean ± standard deviation (SD). Comparisons between two groups were performed using unpaired t-tests (with Welch’s correction applied when variances were unequal). Multiple comparisons among three or more groups were performed using one-way ANOVA followed by Tukey’s multiple comparisons test, or Welch’s ANOVA followed by Dunnett’s T3 multiple comparisons test when variances were unequal. Two-way ANOVA was used to evaluate the effects of two independent variables and their interaction on the measured outcomes; when a significant interaction was detected, Sidak’s multiple comparisons test was performed. *P* value < 0.05 was considered statistically significant. The *Pdcd10*^*fl/fl*^ groups, which did not develop CCM lesions, served exclusively as negative controls to verify the establishment of the CCM phenotype and were thus excluded from quantitative comparisons of lesion and hemorrhage areas.

## Result

### *DPP4 *is a key mediator linking extracellular stimuli to endothelial migration

**Fig. 1:**
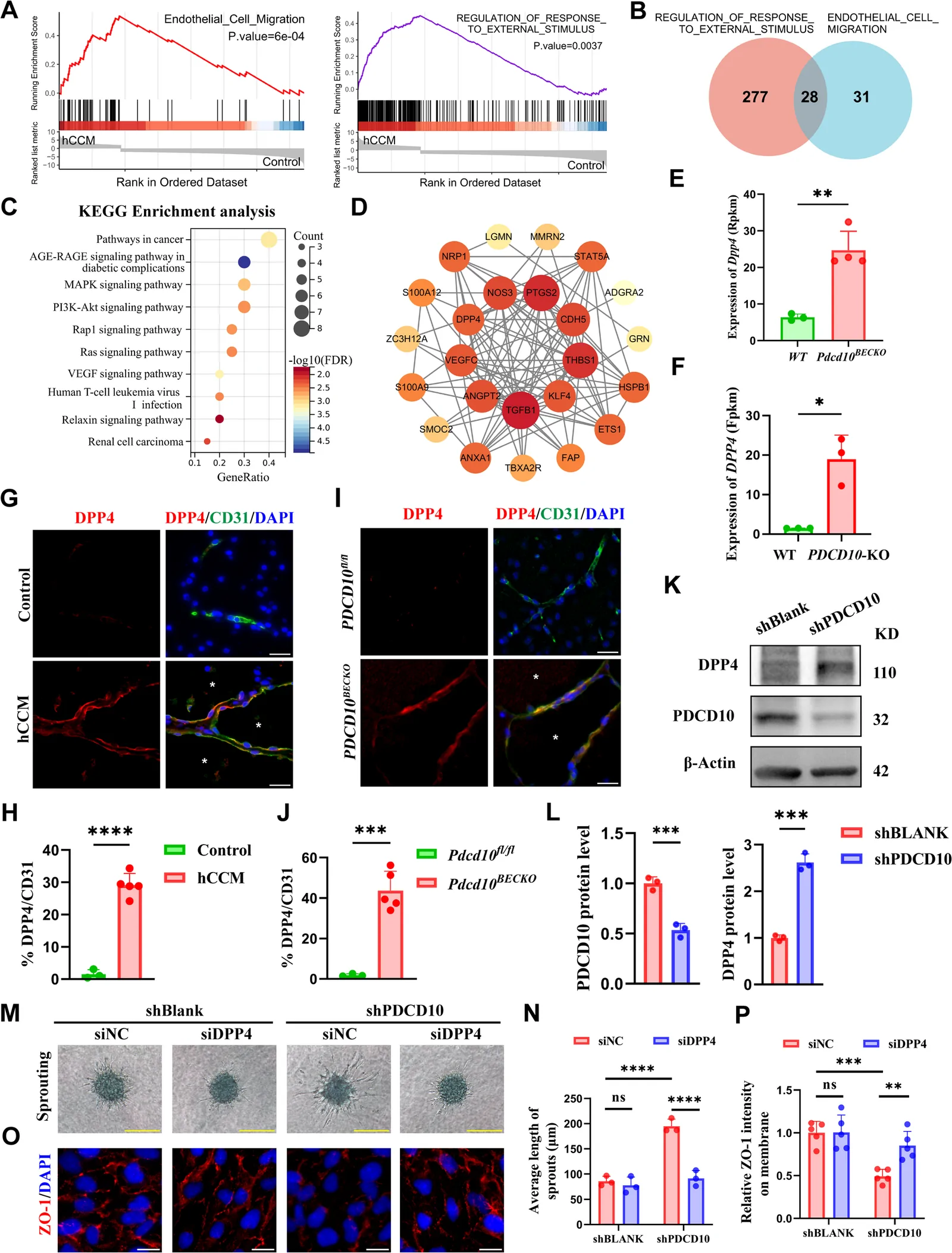
Identify *DPP4* as a key regulator of endothelial dysfunction in CCM. (**A**) GSEA analysis showed that the GOBP_ENDOTHELIAL_CELL_MIGRATION and GOBP_REGULATION_OF_RESPONSE_TO_EXTERNAL_STIMULUS pathways were upregulated in patient CCM lesions. (B) Venn diagram showing common DEGs in the gene sets (**C**) KEGG enrichment analysis of the common DEGs. (**D**) PPI network analysis indicates the hub genes. (**E**) mRNA level of *Dpp4* in *Pdcd10*^BECKO^ primary brain ECs, n = 3 and 4 mice for WT and *Pdcd10*^BECKO^ groups, respectively. (**F**) mRNA level of DPP4 in PDCD10 knockout HUVEC, n = 3. (**G**, **H**) Representative IF images and quantification showed DPP4 upregulated in human CCM lesion (*). Scale bar: 20 μm. n = 3 and 5 specimens for Control and hCCM groups, respectively. (**I**, **J**) Representative IF images and quantification showed DPP4 upregulated in *Pdcd10*^BECKO^ mice CCM lesion (*). Scale bar: 20 μm. n = 3 and 5 mice for *Pdcd10*^fl/fl^ and *Pdcd10*^BECKO^ groups, respectively. (**K**, **L**) WB images and quantification of PDCD10 and DPP4 in PDCD10-depleted hCMEC/D3 cells, n = 3. (**M**, **N**) The effect of DPP4 on migration and angiogenesis was evaluated through spheroid sprouting assays and quantitative analysis, n = 3. Scale bar: 200 μm. (**O**, **P**) Representative IF images and quantification of the membrane-localized ZO-1 in hCMEC/D3 cells, n = 5. Scale bar: 20 μm. Data are presented as mean ± SD. ** P < 0.01, *** P < 0.001, **** P < 0.0001, unpaired Student’s t-test for E, H, and L; unpaired Welch’s correction t-test for F and J; two-way ANOVA followed by Sidak’s multiple comparisons test for N and P

Disordered endothelial migration is a hallmark of cerebral cavernous malformations (CCMs) [5]. To identify transmembrane regulators linking extracellular cues to endothelial migration, we performed GSEA on RNA-seq data from CCM patients (GSE137596). Endothelial migration (GO:0043542) and regulation of response to external stimuli (GO:0032101) were significantly enriched in CCM lesions (Fig. 1A). Intersection of DEGs from these pathways yielded 28 shared candidates (Fig. 1B), which were enriched in CCM-related signaling pathways, including PI3K, MAPK, and VEGF (Fig. 1C). Protein–protein interaction (PPI) network analysis highlighted several hub genes (Fig. 1D), some previously implicated in CCM [10, 26, 27]. Among them, DPP4, a transmembrane protein involved in vascular homeostasis, has not been studied in CCM. We confirmed elevated DPP4 expression across independent datasets, including primary brain ECs from *Pdcd10*^*BECKO*^ mice (GSE204979; Fig. 1E) and PDCD10-deficient HUVECs (GSE137425; Fig. 1F). Immunofluorescence (IF) staining further showed increased DPP4 specifically in CCM-lesional ECs in both human samples and *Pdcd10*^*BECKO*^ mice (Figs. 1G–J), suggesting a role in CCM-associated endothelial dysfunction.

To assess its functional significance, we established an in vitro CCM model by knocking down PDCD10 in hCMEC/D3 cells, which led to increased DPP4 expression (Fig. 1K, L). Silencing DPP4 reversed PDCD10 deficiency–induced increases in spheroid sprouting, migration, and tube formation (Fig. 1M–N; Figure S1A–D). Moreover, DPP4 knockdown partially restored the ZO-1 membrane localization (Fig. 1O, P) and reduced Vimentin expression (Figure S1E, F), indicating attenuation of endothelial-to-mesenchymal transition (EndMT; a critical phenotype in CCM [26]). Together, these findings identify DPP4 as a potential mediator of endothelial dysfunction in CCM.

### Knocking down DPP4 inhibits the progression and maturation of CCM lesions

**Fig. 2:**
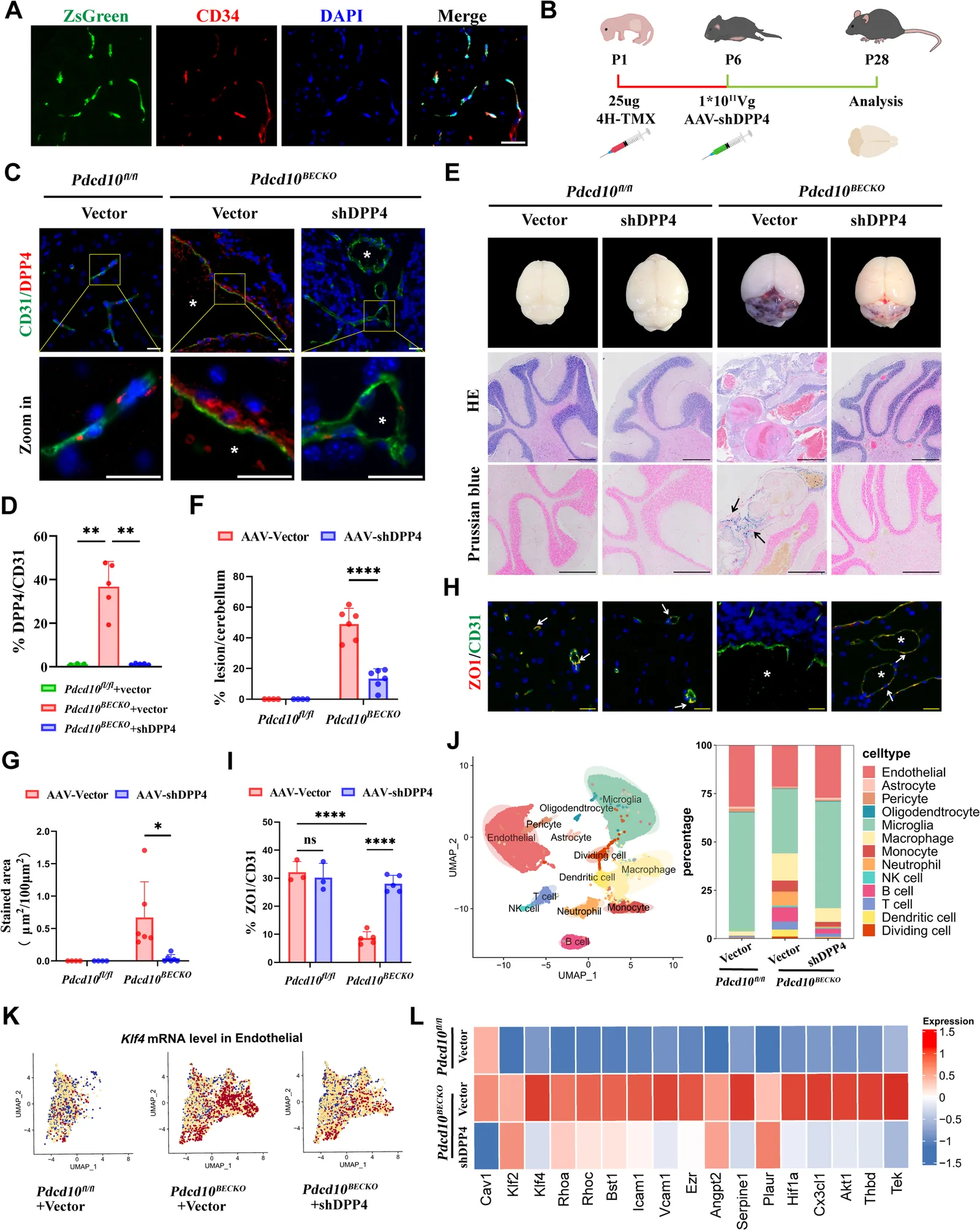
AAV-shDPP4 inhibits CCM progression. (**A**) Representative IF images show that Zsgreen expressed in mouse brain ECs (CD34^+^) ten days after AAV-BI30-Zsgreen administration. (**B**) Schematic diagram of the experimental procedures. (**C**, **D**) Representative IF images and quantification showed that DPP4 was knocked down by AAV-shDPP4 in CCM lesions (*), n = 3, 5, and 5 mice for *Pdcd10*^fl/fl^ +Vector, *Pdcd10*^BECKO^ + Vector, and *Pdcd10*^BECKO^ + AAVshDPP4 groups, respectively. Scale bar: 20 μm. (**E**) Representative images of brain tissue, H&E staining and Prussian blue (black arrows) staining of cerebellum sections. Scale bar: 500 μm. (**F**, **G**) Quantification of lesion area and Prussian blue positive staining area. n = 4, 4, 6, and 6 mice for *Pdcd10*^fl/fl^ +Vector, *Pdcd10*^fl/fl^ + AAVshDPP4, Pdcd10^BECKO^ + Vector, and *Pdcd10*^BECKO^ + AAVshDPP4 groups, respectively. (**H**) Representative IF image of ZO1 is used to evaluate the effect of DPP4 on the endothelial barrier. White arrows indicated normal ZO1 coverage. Scale bar: 20 μm. (**I**) Quantification of ZO1 coverage on CD31^+^ ECs, n = 3, 3, 5, and 5 mice for *Pdcd10*^fl/fl^ +Vector, *Pdcd10*^fl/fl^ + AAVshDPP4,* Pdcd10*^BECKO^ + Vector, and *Pdcd10*^BECKO^ + AAVshDPP4 groups, respectively. (**J**) UMAP (left) displays the cell clusters in scRNA-seq, with cells colored according to the identified clusters. Grotta Bars (right) display the proportion of various cell subpopulations in each group of mice. (**K**) UMAP displays the expression level of *Klf4* in ECs, with redder colors indicating higher expression levels. (**L**) Heat maps display changes in the expression of CCM marker genes in ECs. Data are presented as mean ± SD. ** P < 0.01, **** P < 0.0001, Welch’s ANOVA followed by Dunnett’s T3 multiple comparisons for D; unpaired Student’s t-test for F; unpaired Welch’s correction t-test for G; two-way ANOVA followed by Sidak’s multiple comparisons test for I

To define the role of DPP4 in CCM progression, we generated a brain EC–targeted AAV encoding *Dpp4* shRNA (AAV-shDPP4) using the AAV-BI30 backbone [28], with ZsGreen as a reporter. Ten days after retro-orbital delivery, ZsGreen signal was largely confined to brain microvascular ECs (Fig. 2A). In the acute CCM model, *Pdcd10*^*BECKO*^ mice induced at P1 were treated with AAV-shDPP4 at P6 (Fig. 2B). IF staining confirmed efficient DPP4 silencing in lesional ECs (Fig. 2C, D). This was accompanied by a marked reduction in lesion burden, as shown by decreased lesion area on H&E staining and reduced chronic hemorrhage on Prussian blue staining (Fig. 2E–G). Endothelial junction integrity was also improved. Whereas CCM lesions showed disrupted architecture and loss of ZO-1, AAV-shDPP4 restored ZO-1 localization (Fig. 2H, I), consistent with enhanced barrier function. In parallel, EndMT markers (Vimentin, S100A4) and proliferation marker (KI67) were reduced following DPP4 knockdown (Figure S2A–E).

To assess molecular changes at single-cell resolution, we performed scRNA-seq on *Pdcd10*^*BECKO*^ brains with or without AAV-shDPP4. ECs and immune cells were isolated by flow cytometry (Figure S3A). Analysis of 45,338 cells identified 13 major cell populations, including vascular and immune components (Figs. 2J, S3B). The CCM driver *Klf4* [3] was minimally expressed in control *Pdcd10*^*fl/fl*^ mice ECs but strongly induced in *Pdcd10*^*BECKO*^ mice; this induction was substantially attenuated by AAV-shDPP4 (Fig. 2K). Other CCM-associated genes showed similar trends, including *Cav1*, a known DPP4-interacting protein [17, 18, 29] (Fig. 2L). Consistent with prior reports, *Pdcd10*^*BECKO*^ mice displayed prominent immune infiltration, including neutrophils, B cells, and T cells [30, 31]. DPP4 silencing reduced the proportion of circulating immune cells (Figure S3C–E) and limited microglial transition into a disease-associated state marked by *Apoe*, *Lpl*, and *Spp1* expression (Figure S3F). In addition, macrophages exhibited increased expression of M2 markers (Mrc1, Cd163) (Figure S3G).

Together, these data show that endothelial DPP4 knockdown restrains CCM lesion growth, restores vascular integrity, and dampens neuroinflammatory responses.

### Endothelial subpopulation and CCM-specific trajectories analysis

**Fig. 3:**
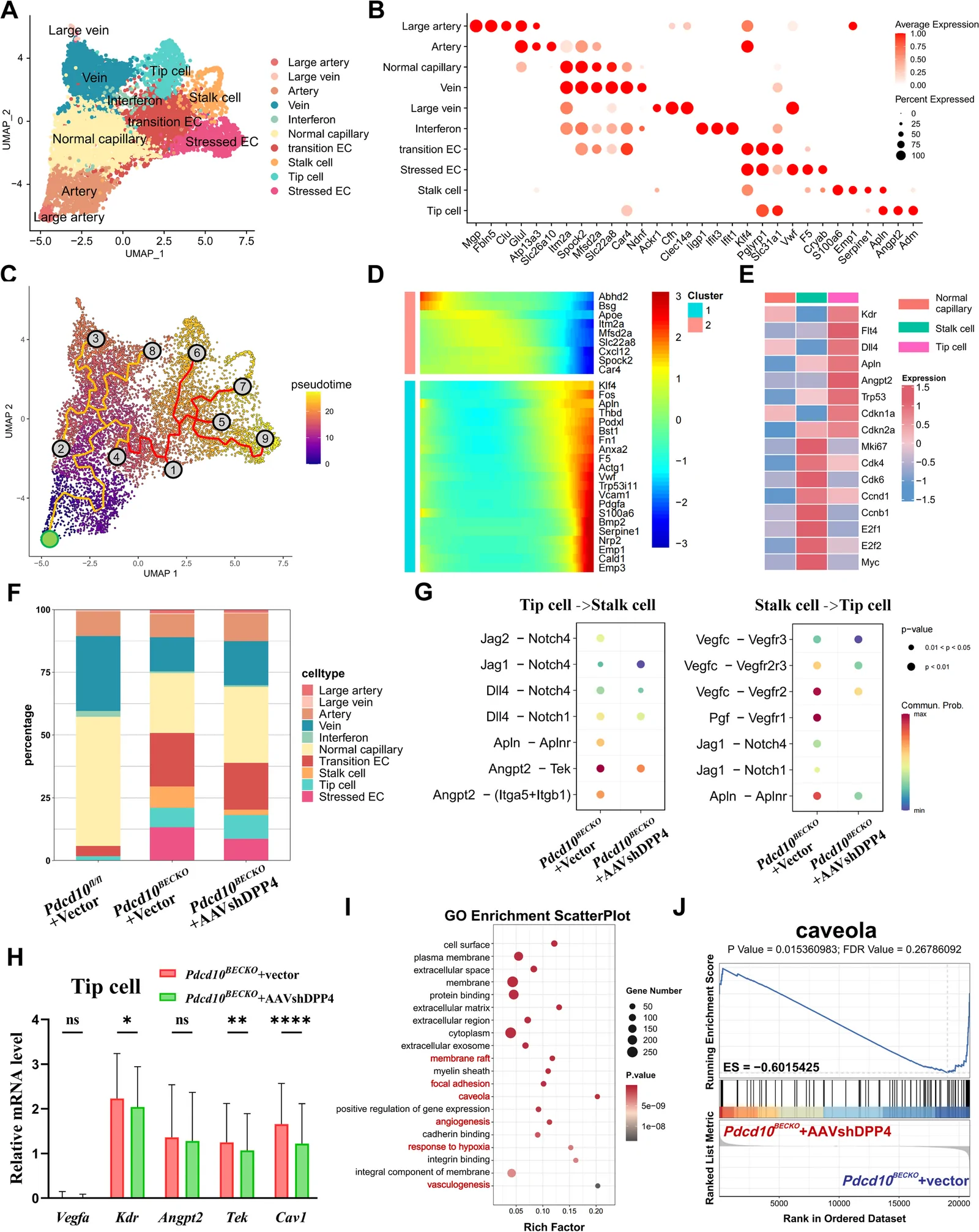
ECs subpopulation analysis and intercellular communication analysis. (**A**) UMAP was used for subpopulation analysis of ECs, with cells colored according to the identified clusters. (**B**) Dot plots for specific marker genes in each cluster. (**C**) Pseudo-time series analysis displays the developmental trajectory of ECs. The color of the points ranges from purple to yellow, representing early to late differentiation. Green circle indicates the origin, and gray circles indicate different cell fates. The red curve represents the approximate trajectory of CCM-specific ECs. (**D**) Heatmap displays the 30 highly variable genes. Red indicates high expression, and blue indicates low expression. (**E**) Heatmap shows the expression levels of specific markers for tip cells and stalk cells. (**F**) Grotta Bars showing the proportion of each EC subpopulation. (**G**) Dot plots for downregulated ligand-receptor pairs between tip cells and stalk cells, the y-axis represents the receptor-ligand relationship pairs. The color indicates the probability of communication, and the size of the points indicates the P-value. (**H**) Quantification of mRNA levels for angiogenesis-related ligands and receptors in tip cells. (**I**) GO enrichment analysis for the DEGs in CCM-specific ECs of AAV-shDPP4-treated versus vector-treated *Pdcd10*^BECKO^ mice. Red labels highlight pathways of interest. (**J**) GSEA analysis shows that “caveola” signaling pathway is downregulated in AAV-shDPP4-treated mice. Data are presented as mean ± SD. * P < 0.05, ** P < 0.01, **** P < 0.0001, unpaired student’s t-test for H

To dissect how DPP4 shapes endothelial signaling in CCM, we subclustered ECs from our scRNA-seq dataset. Among 9,309 ECs, we identified six conserved subtypes (large artery, large vein, artery, vein, interferon‑responsive, and capillary [32]) and four CCM-enriched states: transitional ECs (*Klf4*^*high*^), tip cells (*Angpt2*^*high*^, *Apln*^*high*^), stalk cells (*Emp1*^*high*^, *S100a6*^*+*^), and stressed ECs (*Vwf*^*high*^, *F5*^*+*^) (Fig. 3A, B). These CCM-specific populations were largely restricted to *Pdcd10*^*BECKO*^ mice (Figure S4A). Pseudo-time analysis resolved two trajectories: a canonical vascular path and a CCM-associated branch emerging from capillaries that gives rise to transitional, tip, stalk, and stressed ECs (Fig. 3C). Along this branch, genes linked to thrombosis and stress (e.g.,* Vwf*,* Thbd*,* Fos*,* Vcam1*) and morphogenesis (e.g.,* Klf4*,* Bmp2*,* Bst1*,* Apln*) were upregulated, whereas barrier-associated genes (e.g.,* Mfsd2a*,* Car4*,* Slc22a8*) were reduced (Fig. 3D), consistent with a leaky vascular phenotype.

### scRNA-seq reveals the role of DPP4 in CCM angiogenesis

Tip and stalk cells cooperate to control the development of the vascular network [33]. In the present study, tip cells displayed high expression of angiogenic molecules (e.g.,* Kdr*,* Flt4*,* Angpt2*), while stalk cells were enriched for proliferation markers such as *Mki67* (Fig. 3E). In addition, stalk cells displayed increased *Plvap* (a leakage marker) and reduced tight junction proteins, consistent with a more permeable endothelial phenotype (Figure S4B). DPP4 knockdown reduced the abundance of stalk cells and *F5*^*+*^ stressed ECs, indicating that DPP4 contributes to pathological microenvironment remodeling in CCM (Fig. 3F, Figure S4C).

Although DPP4 knockdown had limited impact on tip cell abundance, it significantly altered endothelial functional coordination. Specifically, cell-cell communication analysis revealed reduced endothelial interaction strength following DPP4 knockdown, especially in pro-angiogenic pathways (Figure S4D, S4E). Notably, ANGPT2-, VEGF-, and Notch-mediated interactions between stalk and tip cells were diminished (Fig. 3G), accompanied by reduced expression of key angiogenic receptors in tip cells, consistent with attenuated pro-angiogenic signaling (Fig. 3H). GO enrichment analysis further showed that DEGs induced by DPP4 silencing were significantly enriched in “*focal adhesion*”, “*angiogenesis*”, “*response to hypoxia*”, and numerous membrane-related signaling pathways (Fig. 3I). Notably, the “*caveolae*” pathway, associated with CAV1-enriched membrane microdomains [18], was significantly downregulated upon DPP4 knockdown (Fig. 3J). Since CAV1 mediates VEGF and ANGPT2 pathways [19, 34–36], it may serve as a key mediator of DPP4-driven pro-angiogenic signaling.

Collectively, these results suggest that DPP4 mediates CCM angiogenesis by altering endothelial cell states and modulating intercellular pro-angiogenic communication, potentially through CAV1-associated membrane signaling platforms.

### DPP4 modulates endothelial dysfunction depending on its promotion in CAV1 phosphorylation

**Fig. 4:**
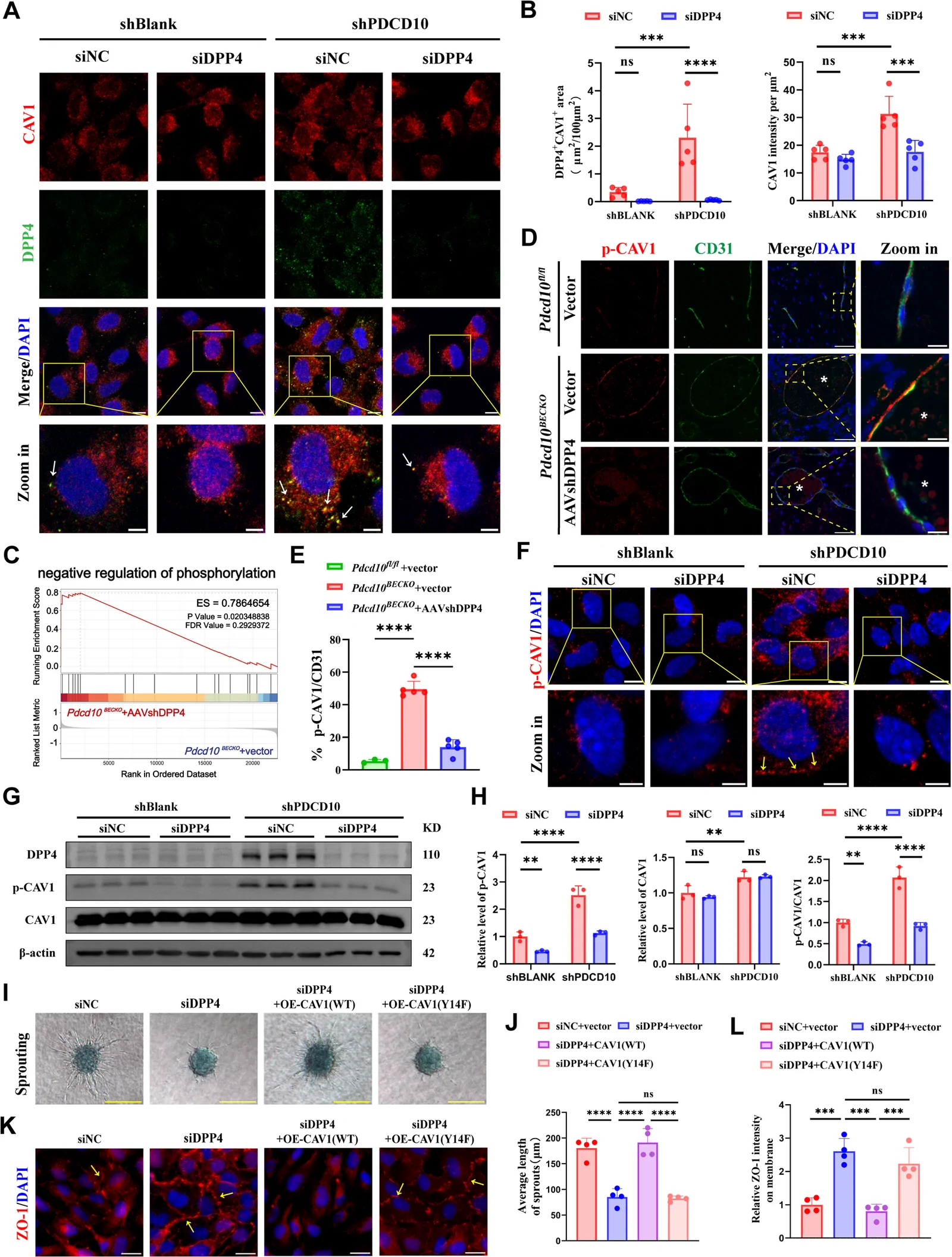
DPP4 promotes endothelial dysfunction in CCM by facilitating CAV1 phosphorylation. (**A**) Representative IF images for the colocalization (arrow) of DPP4 and CAV1 in hCMEC/D3 cells, Scale bar: 10 μm (upper); 5 μm (lower). (**B**) Quantification of CAV1 and DPP4 colocalization (left); quantification of CAV1 fluorescence intensity (right), n = 5. (**C**) GSEA shows that negative regulation of phosphorylation (GO:0042326) pathway was upregulated by DPP4 knockdown in CCM-specific ECs. (**D**) Representative IF images demonstrate increased p-CAV1 in CCM lesions (*), which is suppressed by AAV-shDPP4. Scale bar: 50 μm (left); 10 μm (right). (**E**) Quantification of p-CAV1 coverage on CD31^+^ ECs, n = 3, 5, and 5 mice for *Pdcd10*^fl/fl^ +Vector, *Pdcd10*^BECKO^ + Vector, and *Pdcd10*^BECKO^ + AAVshDPP4 groups, respectively. (**F**) Representative IF images for p-CAV1 expression in hCMEC/D3 cells, arrows indicate p-CAV1 localized to the membrane. Scale bar: 10 μm (upper); 5 μm (lower). (**G**, **H**) WB analysis was used to assess the effect of DPP4 on CAV1 phosphorylation, n = 3. (**I**, **J**) Spheroid sprouting assays and quantification show that the reduced sprouting ability caused by siDPP4 was restored by overexpression of CAV1(WT) but not by CAV1(Y14F), n = 4. scale bar: 200 μm. (**K**, **L**) Representative IF images and quantification show the proper membrane localization of ZO-1 (arrows) caused by siDPP4 was reversed by overexpression of CAV1(WT) but not by CAV1(Y14F), n = 4. scale bar: 20 μm. * P < 0.05, ** P < 0.01, *** P < 0.001, **** P < 0.0001, one-way ANOVA followed by tukey’s multiple comparisons test for E, J and L; two-way ANOVA followed by Sidak’s multiple comparisons test for B and H

CAV1 and caveolae are activated in CCM-lesional ECs, but the upstream trigger is unclear [34]. Phosphorylation of CAV1 at Tyr14 controls its membrane distribution and caveolae internalization [18]. To test whether this modification is engaged in CCM and regulated by DPP4, we examined CAV1 phosphorylation in cellular and in vivo models. IF staining showed increased colocalization of DPP4 and CAV1 in PDCD10-deficient hCMEC/D3 cells (Fig. 4A, B). GSEA suggested that DPP4 knockdown suppresses phosphorylation-related pathways in vivo (Fig. 4C). Consistently, *Pdcd10*^*BECKO*^ mice displayed elevated p-CAV1 in lesional ECs, which was reduced by DPP4 silencing (Fig. 4D, E). At the cellular level, PDCD10-deleption increased membrane-associated p-CAV1 (Fig. 4F), and this effect was dependent on DPP4 (Fig. 4G, H). These data link DPP4 to enhanced CAV1 phosphorylation and caveolae activity in CCM.

To define the functional role of p-CAV1, we generated a phosphorylation-deficient mutant CAV1(Y14F) by substituting tyrosine 14 with phenylalanine. In vitro, overexpression of CAV1(WT), but not CAV1(Y14F), restored p-CAV1 levels reduced by DPP4 knockdown (Figure S5A). Functionally, CAV1(WT) rescued endothelial sprouting reduced by DPP4 silencing, whereas CAV1(Y14F) had no effect (Fig. 4I, J). Moreover, CAV1(WT) reversed the recovery of ZO-1 localization induced by DPP4 knockdown, promoting its redistribution to the cytoplasm, while CAV1(Y14F) preserved ZO-1 membrane localization (Fig. 4K, L). CAV1-mediated internalization of ZO-1 is known to disrupt endothelial barrier integrity [37], explaining how DPP4 knockdown restores ZO1 coverage. These findings support a model in which DPP4 contributes to endothelial dysfunction by regulating CAV1 phosphorylation.

### The DPP4/p-CAV1 axis activates p-FAK in CCM progression

**Fig. 5:**
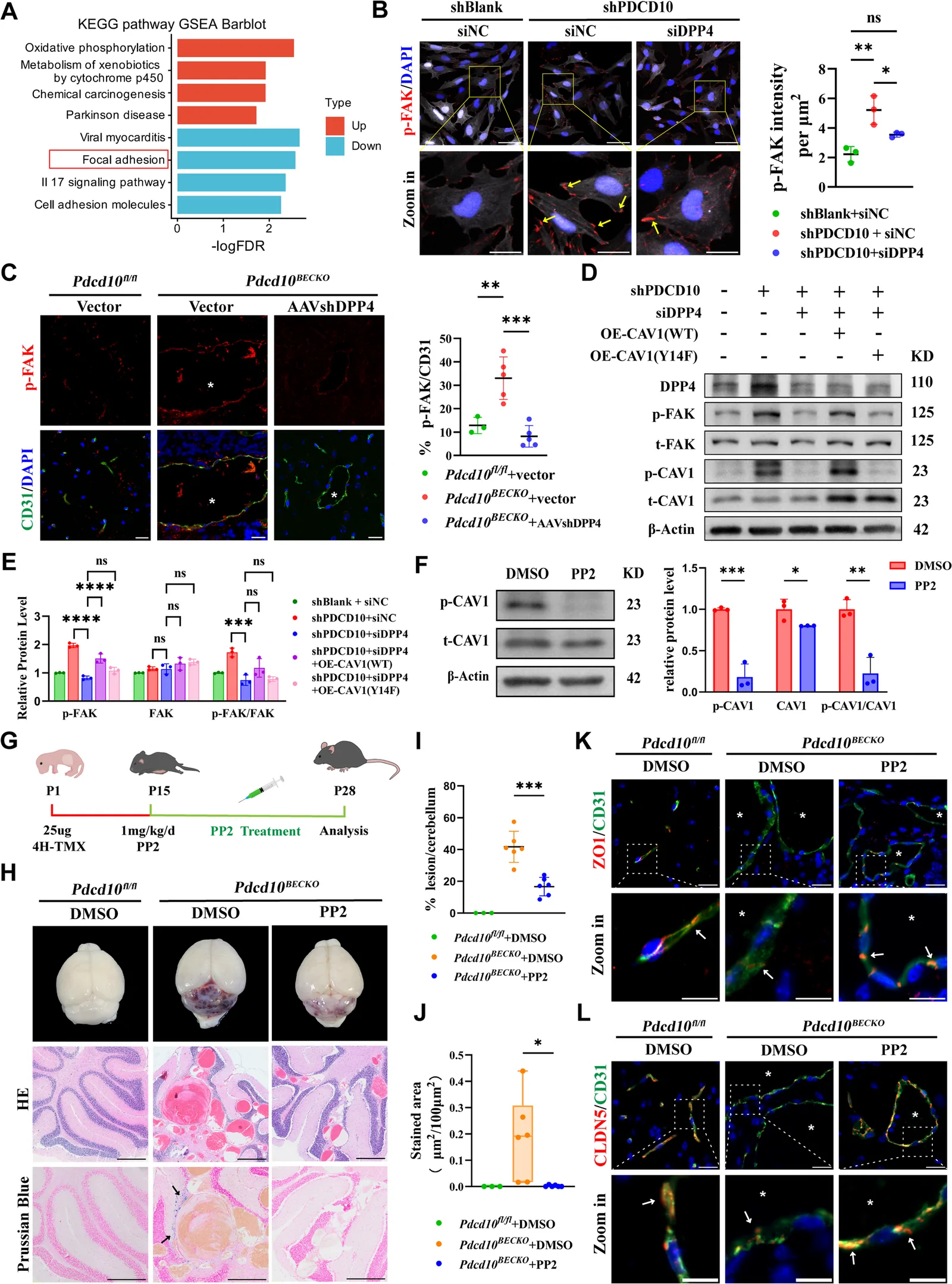
The DPP4/p-CAV1 axis promotes the activation of FAK in CCM progression. (**A**) GSEA analysis of scRNA-seq shows the KEGG pathways affected by AAV-shDPP4 in ECs. Red bars represent upregulate, and blue bars represent downregulation. (**B**) Representative IF images and quantification show that massive p-FAK aggregation (arrows) was reduced by DPP4 knockdown, n = 3. Scale bar: 50 μm (upper); 20 μm (lower). (**C**) Representative IF images and quantification show that p-FAK (red) is inhibited by AAV-shDPP4 in CCM lesions (*). scale bar: 20 μm. n = 3, 5, and 5 mice for *Pdcd10*^fl/fl^ +Vector, *Pdcd10*^BECKO^ + Vector, and *Pdcd10*^BECKO^ + AAVshDPP4 groups, respectively. (**D**, **E**) WB images and quantification show that DPP4 knockdown inhibits p-FAK upregulation. Overexpression of CAV1(WT) reversed this phenomenon, but not CAV1(Y14F), n = 3. (**F**) WB images and quantification for the effect of PP2 on CAV1 phosphorylation in PDCD10-delepted hCMEC/D3 cell, n = 3. (**G**) Schematic diagram of the PP2 administration procedure. (**H**) Representative images of brain tissue, H&E and Prussian blue (arrows) staining. Scale bar: 500 μm. (**I**, **J**) Quantification of the lesion area and Prussian blue positive area, n = 3, 6, and 6 mice for *Pdcd10*^fl/fl^ +DMSO, *Pdcd10*^BECKO^ + DMSO, and *Pdcd10*^BECKO^ + PP2 groups, respectively. Data are presented as mean ± SD (panel **I**) or as box plots (panel J). (**K**, **L**) Representative IF images show that PP2 restores the expression of ZO-1 (up) and Claudin-5 (down) in CCM lesions (*). Arrows highlight ZO-1 or Claudin-5 between ECs. Scale bar: 20 μm (whole); 10 μm (zoom). Data are presented as mean ± SD. * P < 0.05, ** P < 0.01, *** P < 0.001, **** P < 0.0001, one-way ANOVA followed by tukey multiple comparisons test for B, C and E; unpaired Student’s t-test for F and I; unpaired Welch’s correction t-test for J

To identify the downstream pathways affected by DPP4 in CCM, we performed GSEA on scRNA-seq data. Consistent with our earlier GO enrichment analysis, “*Focal adhesion”* pathway was downregulated by DPP4 knockdown with high statistical significance (Fig. 5A). Focal adhesion signaling is a key determinant of endothelial migration and angiogenesis, with FAK acting as a central integrator of inputs from VEGF, RhoA/ROCK, and PI3K pathways [38, 39]. Given that FAK signal transduction depends on CAV1 [40], we examined whether the DPP4/p-CAV1 axis controls p-FAK signaling in CCM.

In human CCM specimens, p-FAK levels were markedly elevated in lesional ECs (Figure S5C, D). In PDCD10-deficient hCMEC/D3 cells, IF staining revealed increased p-FAK with pronounced membrane localization (particularly at cellular protrusions), which was substantially attenuated by DPP4 knockdown (Fig. 5B). This rescue phenotype was recapitulated in *Pdcd10*^*BECKO*^ mice treated with AAV-shDPP4 (Fig. 5C). Western blot analysis confirmed that DPP4 knockdown suppresses p-FAK activation induced by PDCD10 deficiency. Notably, CAV1(WT) overexpression rescued the suppression of p-FAK, while the phosphorylation-deficient mutant CAV1(Y14F) failed to restore signaling (Fig. 5D, E). These data demonstrate that DPP4 regulates p-FAK activation primarily through p-CAV1.

To investigate the role of the p-CAV1/p-FAK axis in CCM progression, we used the selective Src kinase inhibitor PP2, a pharmacological tool commonly used to inhibit CAV1 phosphorylation [41]. Since the FAK phosphorylation requires Src kinase activity, we evaluated PP2’s dual inhibition of both targets. Western blot analysis confirmed PP2 simultaneously suppressed phosphorylation of both CAV1 and FAK (Fig. 5F, S5E). Meanwhile, PP2 also inhibited endothelial migration (Figure S5F). As expected, the PP2 treatment significantly reduced the CCM lesion burden and hemorrhage in the *Pdcd10*^*BECKO*^ mice, although inter-individual heterogeneity in lesion development was observed (Figs. 5G–J). Furthermore, IF staining demonstrated restoration of physiological punctate distributions for ZO-1 and Claudin-5 at endothelial junctions in CCM lesions by PP2 (Fig. 5K, L), underscoring the therapeutic potential of targeting the p-CAV1/p-FAK signaling.

### DPP4 aggravates CCM progression through its non-enzymatic function

**Fig. 6:**
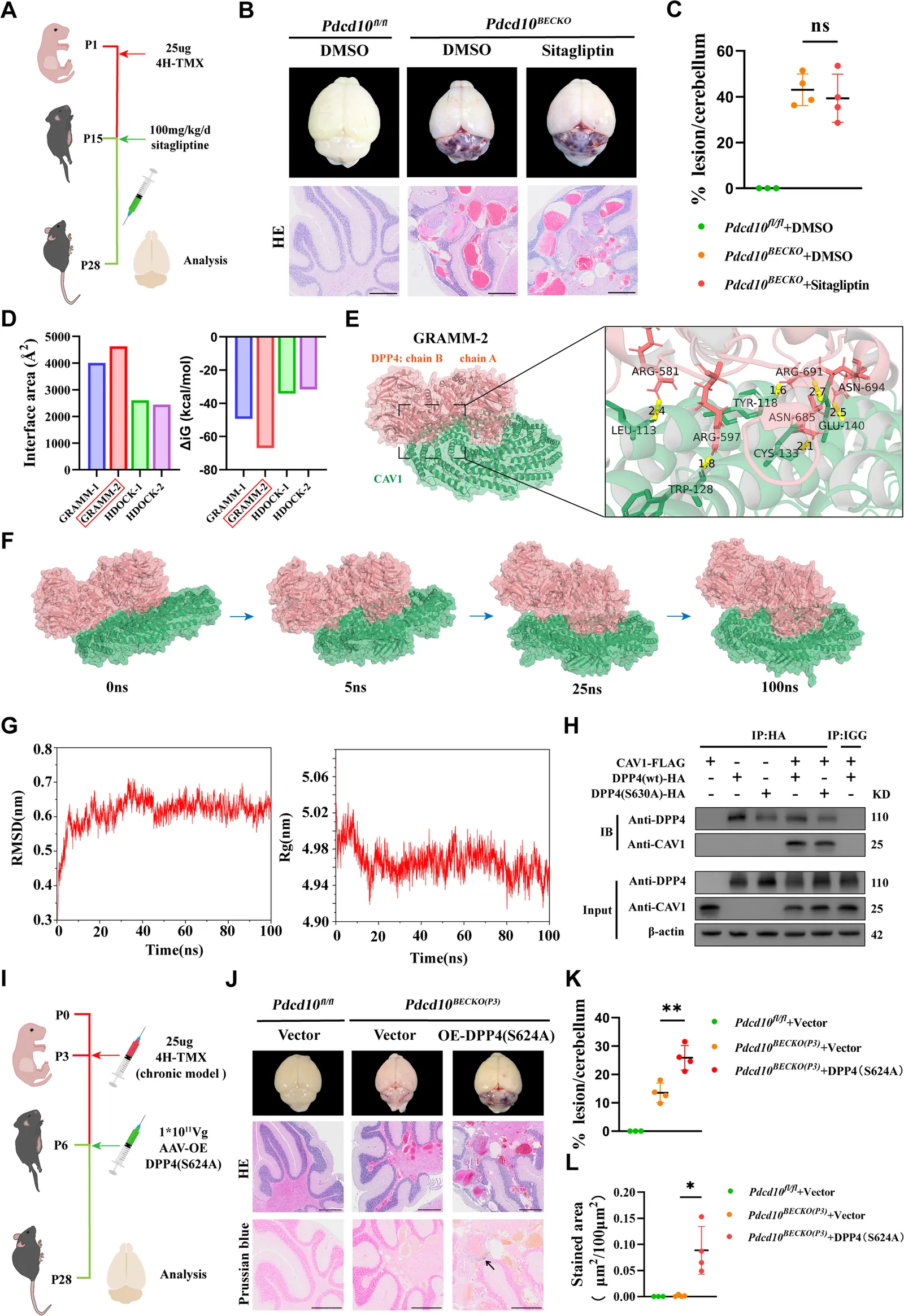
Non-enzymatic interaction of DPP4–CAV1 drives CCM progression. (**A**) Schematic diagram of the sitagliptin administration procedure. (**B**) Representative images of brain tissue and H&E staining. Scale bar: 500 μm. (**C**) Quantification of the proportion of lesion area to cerebellum area. n = 3, 4, and 4 mice for *Pdcd10*^fl/fl^ + DMSO, *Pdcd10*^BECKO^ + DMSO, and Pdcd10BECKO + Sitagliptin groups, respectively. (**D**) The buried interface area and ΔiG of the DPP4-CAV1 complex. (**E**) Structure analysis of the molecular docking shows the docking posture and the hydrogen bonds formed between DPP4 and CAV1. Pink represents DPP4, green represents CAV1. (**F**) Molecular conformation snapshots at different time points during the molecular dynamic simulation. (**G**) Time-dependent RMSD and Rg changes of the complex during molecular dynamics simulation. (**H**) Co-IP experiments reveal that both wild-type DPP4 and enzyme activity-deficient mutant bind to CAV1. (**I**) Schematic diagram of the OE-DPP4(S624A) administration procedure. (**J**) Representative images of brain tissue, H&E staining and Prussian blue (arrows) staining of cerebellum sections. Scale bar: 500 μm. (**K**, **L**) Quantification of lesion area and Prussian blue positive staining area, n = 3, 4, and 4 mice for *Pdcd10*^fl/fl^ + Vector, *Pdcd10*^BECKO(P3)^ + Vector, and *Pdcd10*^BECKO(P3) ^+ OE-DPP4(S624A) groups, respectively. Data are presented as mean ± SD. * P < 0.05, ** P < 0.01, unpaired Student’s t-test for C and K; unpaired Welch’s correction t-test for L

DPP4 exhibits both enzymatic and non-enzymatic functions; however, which function mediates its role in CCM remains unclear. To assess this, we inhibited DPP4 enzymatic activity with sitagliptin in the acute CCM model [12]. Sitagliptin did not affect lesion burden (Fig. 6A–C) and failed to reduce p-FAK levels in PDCD10-deficient hCMEC/D3 cells (Figure S6A, B), suggesting that DPP4-driven signaling in CCM is largely independent of its enzymatic activity.

To further explore the underlying mechanism, we analyzed the structural interaction between DPP4 and CAV1. In its native state, DPP4 forms homodimers [42], while CAV1 assembles into a disc-shaped homomultimeric embedded in the membrane [43–45]. Protein–protein docking using two tools (HDOCK and GRAMM) yielded highly consistent top-ranked conformations (Fig. 6E, S6C). Among these, the GRAMM-2 model exhibited the largest interface and most favorable solvation energy (Fig. 6D). In this model, residues R581, R597, N685, R691, and N694 of the DPP4 B-chain form an extensive hydrogen-bond network with CAV1, while the A-chain interacts with the central hydrophobic β-barrel of CAV1 (Fig. 6E, Figure S6D). To evaluate the stability of the GRAMM-2 model, we performed a 100 ns molecular dynamics simulation. Structural snapshots from this trajectory illustrated the conformational dynamics over time and suggested that CAV1 underwent conformational compaction upon DPP4 binding, forming a concave architecture that may provide structural insights into the potential regulation of caveola-associated processes (Fig. 6F) [45]. Notably, after 10 ns, the RMSD reached equilibrium and the Rg value decreased, indicating a more compact structure upon binding (Fig. 6G). The energy landscape displayed a single minimum, consistent with a stable conformation (Figure S6E). These observations collectively support the stability of this complex.

Importantly, the CAV1-interacting residues of DPP4 are surface-exposed and spatially distinct from the buried catalytic site (S630–D708–H740) [46] (Figure S7A). To directly test whether enzymatic activity is required, we generated an enzymatically inactive human DPP4 mutant (S630A) and, in parallel, constructed AAV vectors to overexpress the corresponding mouse mutant (S624A) in vivo [14]. Co-IP assays showed that DPP4 (S630A) retained its ability to bind CAV1 (Fig. 6H). Consistently, AAV-mediated overexpression of DPP4 (S624A) significantly increased lesion burden and hemorrhage in the chronic CCM model, although biological variability was observed among individual mice. (Fig. 6I–L). Collectively, these results indicate that DPP4 promotes CCM progression predominantly in an enzymatic activity–independent manner, likely through its interaction with CAV1.

### Targeting the DPP4–CAV1 interaction suppresses CCM progression

**Fig. 7:**
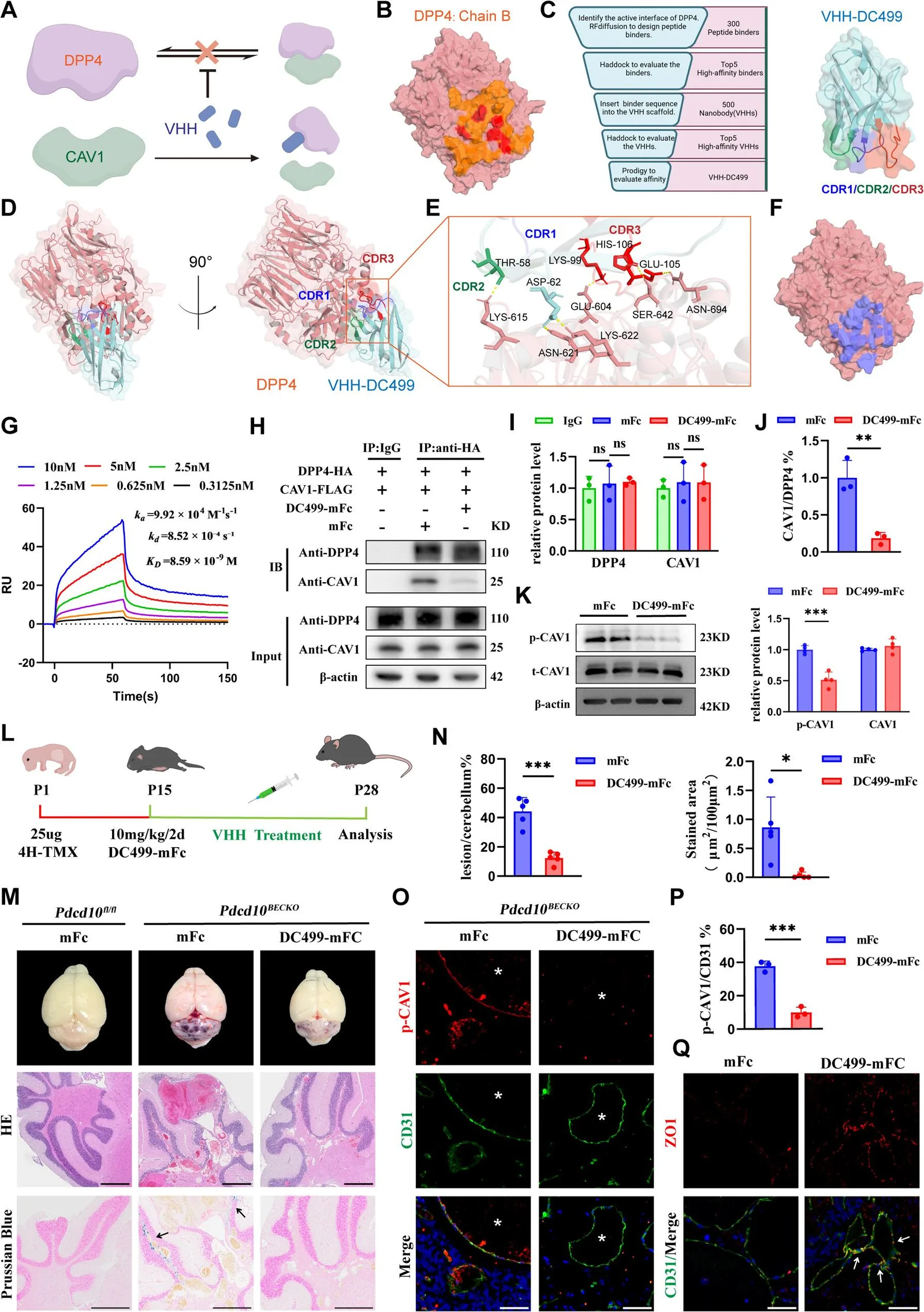
The engineered nanobody DC499-mFc disrupts the DPP4–CAV1 interaction and inhibits CCM progression. (A) Schematic of VHH blocking DPP4–CAV1 interaction. (**B**) Key amino acids (red) and hotspot regions (orange) for DPP4 binding to CAV1. (**C**) Screening process for VHH-DC499, and Alphafold3-predicted structure (right) of DC499. (**D**) Molecular docking of VHH-DC499 binding to DPP4. (**E**) Hydrogen bonds formed between VHH-DC499 and DPP4. (**F**) Coverage of VHH-DC499 on the DPP4 surface (purple). (**G**) The SPR experimental analysis revealed that DC499-mFc specifically binds to DPP4, with a KD value of 8.59×10-9 M. (**H**) Co-IP indicates that DC499-mFc blocked the DPP4-CAV1 interaction. (**I**) Quantification of the input DPP4 and input CAV1, n=3. (**J**) Quantitation for the proportion of CAV1 binding to DPP4, n=3. (**K**) WB analysis revealing that DC499-mFc significantly inhibits p-CAV1 in PDCD10-depleted hCMEC/D3 cells, n=4. (**L**) Schematic diagram of the DC499-mFc administration procedure. (**M**) Representative images of brain tissue, H&E staining and Prussian blue (arrows) staining of cerebellum sections. Scale bar: 500 μm. (**N**) Quantification of lesion area and Prussian blue positive staining area, n=5. (**O**, **P**) Representative IF images and quantification show that p-CAV1 is suppressed by DC499-mFc in CCM lesions (*), n=3. Scale bar: 50μm. (**Q**) Representative IF images show that DC499-mFc restores the physiological distribution of ZO-1 (arrows) in CCM lesions, Scale bar: 50μm. Data are presented as mean ± SD. * P<0.05, ** P<0.01, *** P<0.001, one-way ANOVA followed by tukey multiple comparisons test for I; unpaired Student’s t-test for J, K, N(left) and P; unpaired Welch’s correction t-test for N(right).

To disrupt the DPP4–CAV1 interaction, we first evaluated clinically approved DPP4 inhibitors, including sitagliptin, linagliptin, and teneligliptin. These small-molecule inhibitors target the catalytic site of DPP4 but do not engage the predicted CAV1-binding interface (Figure S7B). Given the extracellular accessibility of DPP4, we pursued a nanobody-based strategy (Fig. 7A-C). Nanobodies (VHHs) are well suited to block protein–protein interactions by sterically occluding extended interfaces, owing to their small size and structural adaptability [47]. Importantly, unlike conventional nanobody screening approaches that lack spatial precision in epitope targeting, we employed a structure-guided, AI-driven strategy to achieve selective recognition of pre-defined structural hotspots on DPP4.

First, using the generative AI tool RFdiffusion, we *de novo* designed peptide binders to specifically target the predicted CAV1-binding interface on DPP4. The top five peptide candidates, ranked by HADDOCK3 scores (Figure S7C), were grafted into the CDR3 of a VHH scaffold. Subsequent random mutagenesis of CDR1 and CDR2 yielded a VHH variant library [24]. The VHH variants were prioritized using a multi-parameter in silico pipeline, including docking score, predicted binding affinity, and interface complementarity. Among these, VHH-DC499 emerged as the top candidate based on its superior interface complementarity and predicted binding affinity (Figure S7D, E). Structural modeling indicated strong surface complementarity between VHH-DC499 and DPP4, with a hydrogen-bonding network formed by VHH-DC499 residues Asp62 (framework), Thr58 (CDR2), and Lys99, His106, and Glu105 (CDR3) interacting with DPP4 (Fig. 7D, E). Importantly, structural modeling predicts that VHH-DC499 occupies the CAV1-binding groove on DPP4 (Fig. 7F), supporting its potential to interfere with the DPP4–CAV1 interaction.

To enhance in vivo stability, VHH-DC499 was fused to the Fc fragment of mouse IgG1, and a dimeric construct (DC499-mFc) was generated by exploiting the dimerization property of the Fc domain. DC499-mFc was efficiently expressed in CHO-S cells, with a protein yield of 604.8 mg/L. SDS-PAGE analysis revealed a predominant band at approximately 80 kDa under non-reducing conditions and a major band at approximately 40 kDa under reducing conditions, consistent with Fc-mediated disulfide-linked dimerization (Figure S7F). The calculated molecular weights of the monomer (38.7 kDa) and dimer (77.4 kDa) were consistent with the observed biochemical characteristics of DC499-mFc. Importantly, SEC-HPLC analysis further demonstrated a single symmetrical peak with a purity of 97.21%, indicating a stable and monodisperse dimeric form of DC499-mFc under native conditions (Figure S7G).

The binding affinity of DC499-mFc to DPP4 was evaluated using SPR. The sensorgrams showed a concentration-dependent increase in response units (RUs), supporting the specific interaction between DC499-mFc and DPP4 (Fig. 7G). Kinetic analysis of the SPR sensorgrams yielded an equilibrium dissociation constant (*K*_*D*_) of 8.59 × 10^− 9^ M, indicative of high-affinity binding. To further corroborate the binding in solution, MST was performed and yielded a *K*_*D*_ value of 5.37 ± 0.67 × 10^− 9^ M (Figure S7H). The close agreement between SPR and MST measurements collectively demonstrates the favorable binding affinity of DC499-mFc for DPP4. Immunoprecipitation confirmed DC499-mFc binding to endogenous DPP4 in hCMEC/D3 lysates, whereas the Fc control showed no interaction (Figure S7I). Functionally, DC499-mFc disrupted the DPP4–CAV1 interaction and reduced CAV1 phosphorylation in vitro (Fig. 7H–K).

Human and mouse DPP4 are highly conserved, including the predicted CAV1-binding interface (Figure S7J). Docking analyses indicated that VHH-DC499 binds to mouse DPP4 in a similar mode as to human DPP4 (Figure S7K, L). Pharmacokinetic analysis demonstrated that, following a single intraperitoneal administration of DC499-mFc at 10 mg/kg, C_max_ reached 96.237 ± 10.256 µg/mL at 2 h post-administration, and t_1/2_z was 36.975 ± 6.036 h. The calculated pharmacokinetic parameters indicated prolonged systemic exposure and low clearance of DC499-mFc, supporting its favorable pharmacokinetic profile (Figure S8A). Repeated dosing at 10 mg/kg every two days for two weeks did not significantly alter body weight compared with the control group (Figure S8B), and no obvious damage to major organs was observed (Figure S8C).

Following in vivo administration, IF staining revealed that DC499‑mFc was markedly enriched in ECs within CCM lesions, whereas lower signal was detected in normal ECs (Figure S8D), indicating its preferential targeting of CCM-lesional ECs. Notably, administration of DC499-mFc significantly reduced lesion area and hemorrhage in the acute CCM model (Fig. 7L–N). IF staining further showed decreased CAV1 phosphorylation and restoration of ZO-1 at endothelial junctions (Fig. 7O–Q). Together, these results support functional disruption of the DPP4–CAV1 interaction as a viable therapeutic strategy for CCM.

## Discussion

CCMs are characterized by endothelial dysfunction and recurrent bleeding, leading to severe neurological consequences [3, 4]. While the genetic drivers of CCM are well established, the specific transmembrane signal mediators that integrate extracellular cues to endothelial dysfunction have remained elusive. In this study, we identified the transmembrane glycoprotein DPP4 as a pivotal upstream regulator of endothelial dysfunction in CCM. Crucially, our data indicate that DPP4 drives CCM pathogenesis not through its classical enzymatic activity, but via a non-enzymatic interaction with CAV1. By deploying a structure-guided nanobody (DC499-mFc) that disrupts the DPP4–CAV1 interaction, we significantly reduced CCM progression in vivo, offering a previously unrecognized pharmacological strategy for this intractable disease. This AI-guided nanobody design strategy represents a conceptually distinct and promising approach for targeting protein–protein interaction interfaces, potentially overcoming the limitations of conventional approaches that indiscriminately target entire proteins.

A major conceptual advance of our study is the identification of a non-enzymatic role of DPP4 in CCM-associated vascular pathology. Given that angiogenesis is a central feature of CCM lesion development, the role of DPP4 in vascular remodeling warrants clarification. Previous studies have reported seemingly contradictory functions of DPP4 in angiogenesis. In physiological or reparative angiogenesis, such as myocardial infarction and diabetic wound healing, the enzymatic activity of DPP4 has been shown to suppress angiogenic responses, at least in part through cleavage of the chemokine CXCL12, thereby limiting endothelial recruitment and tissue repair [48, 49]. In other pathological contexts, DPP4 has been shown to promote angiogenesis through modulation of pro-angiogenic signaling pathways [50–52]. These findings collectively suggest that DPP4 exerts context-dependent effects that are not strictly determined by its enzymatic activity. Extending this concept, our data demonstrate that the function of DPP4 in CCM is largely independent of its enzymatic activity. Conventional small-molecule DPP4 enzymatic inhibitor, sitagliptin, which targets the catalytic pocket, failed to attenuate CCM lesion burden, whereas expression of an enzymatically inactive DPP4 mutant in the chronic CCM mouse model exacerbated disease progression.

Extensive evidence indicates that DPP4 is highly responsive to pathological microenvironmental cues. Conditions such as hypoxia and inflammation upregulate DPP4 expression [53–56]. Notably, these factors are key contributors to CCM pathophysiology [7, 8], raising the possibility that DPP4 may act as a key integrator of microenvironmental signals during disease progression. To further define the role of DPP4 in the CCM pathological microenvironment, our scRNA-seq data provide a high-resolution map of how DPP4 perturbs endothelial signal transmission and immune infiltration in CCM lesions. This analysis indicates that DPP4 promotes pathological endothelial reprogramming in CCM, fostering a pro-angiogenic and destabilized vascular phenotype. DPP4 knockdown suppressed these aberrant endothelial states and disrupted the coordinated intercellular signaling required for pathological angiogenesis. In addition, DPP4 knockdown also reshaped the immune microenvironment of CCM, specifically by reducing peripheral immune cell infiltration and preventing the activation of resident microglia. Given that BBB disruption and neuroinflammation are primary drivers of CCM-related hemorrhage and neurological deficits [7, 57, 58], these findings support DPP4 as a central therapeutic target capable of modulating both vascular pathology and neuroinflammatory responses.

The transduction of CAV1 signaling primarily relies on its scaffold domain (CSD, a conserved sequence from residues 82–101) to mediate interactions with downstream protein [43]. Recent cryo-EM structural analyses have revealed that CAV1 exists on the membrane as a disc-shaped multimer, with the CSD positioned buried within the cell membrane, suggesting CSD typically inaccessible for its downstream ligands under resting conditions [43–45]. However, previous studies have shown that the phosphorylation at the Y14 site of CAV1 is crucial for the internalization of CAV1 and the caveolae-dependent signal transduction [40, 59]. In the present study, our data suggest that the DPP4–CAV1 interaction facilitates the phosphorylation of CAV1-Y14, which in turn triggers FAK activation. Subsequently, the p-CAV1/p-FAK axis likely acts as an intracellular “executor,” driving pathological cell migration and activating other pathogenic signaling cascades. As a key downstream protein regulated by p-CAV1, p-FAK plays a crucial role in cell-matrix interactions and the transmission of force signals [59–61]. It is notable that the downstream targets of p-FAK include numerous established pathways related to CCM, such as PI3K-mTOR and RhoA/ROCK [39]. Meanwhile, since the internalization of CAV1 transports tight junction proteins into the cytoplasm [37], this DPP4/p-CAV1 axis may compromise the integrity of the CCM endothelial barrier.

The non-enzymatic mechanism uncovered here dictated a shift in therapeutic design. Compared with small-molecule inhibitors, which struggle to effectively disrupt the relatively flat PPI interfaces, VHHs are uniquely suited for this task due to their small size, high stability, and extended CDR3 loops. These features allow VHHs to penetrate and bind complex steric grooves that are inaccessible to conventional antibodies [47]. In contrast to conventional VHH library construction and screening, which rely on animal immunization, the structural biology–based AI design explicitly accounts for the specific steric architecture of antigenic epitopes [62]. In the present study, our objective was to evaluate whether predefined structural hotspots could support a plausible competitive interaction at the DPP4–CAV1 interface. To this end, we employed a stepwise, structure-guided design strategy in which designs were generated with constraints focused on the predicted hotspots, followed by iterative in silico assessment of binding poses and interface complementarity. The resulting nanobody showed favorable potential binding characteristics toward the target interface. Consistent with this design rationale, administration of DC499-mFc effectively disrupted the DPP4–CAV1 interaction, reduced CAV1 phosphorylation, and alleviated lesion burden and hemorrhage in vivo. Together, these results support the feasibility of combining generative design with structure-based evaluation to target challenging protein–protein interactions.

Despite these promising findings, our study still has certain limitations that warrant future investigation. Firstly, although we used the PDCD10-deficient model, which is the most aggressive animal model, future studies still need to confirm the efficacy of the DPP4–CAV1 in other genetic background models. Moreover, the number of clinical specimens included in this study was relatively small. Therefore, DPP4 expression should be further validated in a larger cohort of patients, particularly those with diverse genetic etiologies and clinical presentations. Second, while our study primarily assessed the impact of disrupting the DPP4–CAV1 interaction on CCM progression, further studies are warranted to fully elucidate the underlying molecular mechanisms by which this interaction regulates CCM pathogenesis. In particular, the specific extracellular signals transduced by this signaling node require further investigation; how the interaction between DPP4 and CAV1 promotes CAV1 phosphorylation remains unclear; and the role of p-FAK activation in CCM progression, along with other upstream and downstream effectors, constitutes a key focus for future studies. Furthermore, although DC499-mFc demonstrated promising therapeutic efficacy in the *Pdcd10*^*BECKO*^ CCM model and exhibited favorable preliminary pharmacokinetic and safety profiles, more comprehensive pharmacodynamic characterization, long-term toxicological evaluation, and validation in additional preclinical models will be required before clinical translation. Finally, exploring the exact mechanism by which extracellular mechanical or biochemical stimuli initially upregulate DPP4 expression in CCM lesions will provide a more complete picture of the disease initiation process.

## Conclusion

Collectively, our findings suggest that the non-enzymatic DPP4–CAV1 interaction promotes endothelial dysfunction and disease progression in CCM. By engineering a structure-guided nanobody (DC499-mFc) that disrupts this physical interaction, we attenuated the lesion growth and barrier disruption of CCM. These results extend the current knowledge of DPP4 biology within the vascular system and simultaneously provide a potential translatable, targeted therapeutic framework for cerebral cavernous malformations. 

## Supplementary Information

Below is the link to the electronic supplementary material.

Supplementary Material 1

## Data Availability

Relevant data are accessible from the corresponding author upon justified request.

## Abbreviations

AAV-shDPP4: Adeno-associated virus expressing short hairpin RNA for DPP4
ANGPT2: Angiopoeitin-2
CAV1(Y14F): Caeolin-1 (tyrosine at site 14 mutant to phenylalanine)
CCM: Cerebral Cavernous Malformation
CreERT2: Tamoxifen-induced Cre Estrogen receptor
CSD: Scaffold Domain of CAV1
DPP4(S630A): human DPP4 (Serine at site 630 mutant to Alanine)
DPP4(S624A): mouse DPP4 (Serine at site 624 mutant to Alanine)
EndMT: Endothelial-to-mesenchymal transition
FAK: Focal adhesion kinase
hCMEC/D3: Human cerebral microvascular endothelial cells
KLF4: Kruppel-like factor 4
KRIT1: Krev/Rap1-interacting trapped 1
MAPK: mitogen-activated protein kinase
MST: Microscale thermophoresis
mTOR: Mechanistic target of rapamycin
PDCD10: Programmed cell death protein 10
Pdcd10^BECKO^: Conditional knockout of Pdcd10 in brain endothelial cells
PI3K: Phosphoinositide 3-kinase
Rhoa: Ras homolog gene family member A
Rg: Radius of gyration
RMSD: Root mean square deviation
ROCK: Rho-associated coiled-coil forming protein kinase
Slco1c1: Solute carrier organic anion transporter family member 1C1
SPR: Surface plasmon resonance
VEGFA: Vascular endothelial growth factor A
Nanobody/VHH: Variable domain of Heavy chain of Heavy-chain-only antibody
