# Supplementary material for: Targeting the non-enzymatic function of DPP4 via a nanobody inhibits cerebral cavernous malformation progression
Source: J Nanobiotechnology. 2026 Aug 5;24:1014. doi: 10.1186/s12951-026-04898-x (PMC13726199; doi:10.1186/s12951-026-04898-x)
Supplement: Supplementary file 1 [file 12951_2026_4898_MOESM1_ESM.docx]

**Supplemental Figures**

**Figure S1.**

**(A, B)** Wound healing assays show that DPP4 knockdown inhibited the migration in the in vitro CCM model, n=3. Scale bar: 200 μm. **(C, D)** Tube formation assays show that DPP4 knockdown reduced the vesssels length in the in vitro CCM model, n=6. Scale bar: 200 μm. **(E, F)** WB analysis and quantification show that PDCD10 knockdown caused increased expression of Vimentin, which was restored by DPP4 knockdown, n=3. Data are presented as mean ± SD. * P<0.05, ** P<0.01, *** P<0.001, **** P<0.0001, unpaired Student’s t-test for B and D, two-way ANOVA followed by Sidak’s multiple comparisons test for F.

**Figure S2.**

**(A)** Representative IF images show that AAV-shDPP4 reduced Vimentin expression in CCM ECs. Scale bar: 20 μm. **(B)** Representative IF images show that AAV-shDPP4 reduces S100A4 expression in CCM ECs. Scale bar: 20 μm. **(C)** Quantification of Vimentin coverage on CD31^+^ ECs (left); quantification of S100A4 coverage on CD31^+^ ECs (right), n=3. **(D)** Immunohistochemical staining shows that AAV-shDPP4 treatment reduces the number of KI67^+^ ECs in CCM lesions, Scale bar: 200 μm (upper); 50 μm (lower). **(E)** Quantification of KI67-positive areas, n=3. Data are presented as mean ± SD. * *P*<0.05, ** *P*<0.01, **** *P*<0.0001, one-way ANOVA followed by Tukey’s multiple comparisons test for C; unpaired Student’s t-test for E.

**Figure S3.**

**(A)** Schematic diagram of scRNA-Seq experimental procedure. **(B)** Volcano plot shows the five significant feature genes of each cluster based on Log2FC. **(C)** Grotta Bars show the proportion of immune cells in each group. **(D, E)** Fan charts show the proportion of immune cells in each group. AAV-shDPP4 reduced the infiltration of peripheral blood immune cells in CCM lesions. **(F)** Heat maps display changes in the expression of inflammatory-related genes in microglia. Red block indicated the upregulated genes, and blue block indicated the downregulated genes. **(G)** Volcano plot displays DEGs in macrophages between AAV-shDPP4-treated and vector-treated Pdcd10BECKO mice. Red indicates upregulation, blue indicates downregulation.

**Figure S4.**

**(A)** UMAP shows that transition EC, tip cell, stalk cell, and stressed cell specifically exhibits in the *Pdcd10^BECKO^* mice. **(B)** Dot plots show that stalk cells highly express *Plvap* and lowly express *Tjp1*, *Cldn5*, and *Ocln*. **(C)** Fan charts show that AAV-shDPP4 reduces the proportion of stalk cell and stressed EC in CCM. **(D)** Endothelial cell-cell interaction comparing AAV-shDPP4-treated and vector-treated groups. Left: interaction counts; Right: interaction strength. **(E)** Pathway information flow differences between groups. Left:Relative information flow between two groups; Right: Pathway information flow strength in each group. Blue labels: AAVshDPP4 group; Red labels:vector group.

**Figure S5.**

**(A, B)** The WB images and quantitative analysis showed that overexpression of CAV1 (Y14F) was unable to restore the downregulation of p-CAV1 caused by DPP4 depletion, n=3. **(C, D)** Representative IF images and quantification show that p-FAK is activate in human CCM-lesional ECs, n=3. Scale bar: 20 μm. **(E)** WB images and quantification for the effect of PP2 on FAK phosphorylation in PDCD10-delepted hCMEC/D3 cell, n=3. **(F)** Wound healing assays and quantitative analysis show that PP2 inhibited endothelial migration in shPDCD10-treated hBMEC/D3 cells; n=3. Scale bar: 200 μm. Data are presented as mean ± SD. * *P*<0.05, ** *P*<0.01, *** *P*<0.001; one-way ANOVA followed by Tukey’s multiple comparisons test for B; unpaired Student’s t-test for D, E and F.

**Figure S6.**

**(A, B)** WB analysis and quantification revealing sitagliptin treatment did not significantly alter p-FAK in PDCD10-depleted hCMEC/D3 cells, n=3. **(C)** The docking posture of DPP4 and CAV1 in other modes, including GRAMM-1, Hdock-1 and Hdock-2. **(D)** Hydrophobic interaction interface between DPP4 and CAV1. Darker shades indicate stronger hydrophobic interactions. **(E)** The Gibbs free energy landscape of the DPP4-CAV1 complex. Data are presented as mean ± SD. ** *P*<0.01, *** *P*<0.001; two-way ANOVA followed by Sidak’s multiple comparisons test for B.

**Figure S7.**

**(A)** The binding interface (orange) and key residues (red) of DPP4 for CAV1 binding; The DPP4 enzyme catalytic triad (blue). **(B)** Sitagliptin, linagliptin and teneligliptin bind to the DPP4 enzyme catalytic domain. **(C)** The HADDOCK scores of the top five binders. **(D)** The HADDOCK scores of the top five VHHs. **(E)** The affinity indexes predicted by PODIGY of the top five VHHs. Left: predicted binding ΔG; Right: predicted Kd at 37℃. **(F)** SDS‑PAGE confirming DC499‑mFc: monomer (~38 kDa) and dimer (~77 kDa) under reducing and non‑reducing conditions, respectively. **(G)** SEC‑HPLC analysis demonstrated a single symmetrical peak with a purity of 97.21% of DC499‑mFc. **(H)** The MST experimental analysis revealed that DC499-mFc specifically binds to DPP4, with a *K_D_* value of 5.37 ± 0.67 × 10^-9^ M. Data are presented as mean ± SD. **(I)** Immunoprecipitation shows that DC499-mFc can bind to DPP4. **(J)** Cross-species sequence alignment of human and mouse DPP4 from aa 550 to 700. Green represents the same amino acids, while red represents the amino acids that are different. **(K)** Molecular docking for VHH-DC499 binds to mouse DPP4. **(L)** The HADDOCK scores and PODIGY predicted affinity indexes of VHH-DC499 binds to huaman and mouse DPP4.

**Figure S8.**

**(A)** Plasma concentration-time profile of DC499‑mFc in P15 mice following a single intraperitoneal injection at 10 mg/kg. Blood samples were collected at 1, 2, 6, 24, 72, and 120 h post‑injection (n = 3 per time point). **(B)** Body weight changes of *Pdcd10^BECKO^* mice receiving intraperitoneal DC499‑mFc every other day from P15 to P28, with PBS‑treated *Pdcd10^fl/fl^* and *Pdcd10^BECKO^* mice as controls (n = 3). **(C)** Represntative H&E‑stained sections of major organs from mice treated with DC499‑mFc every other day from P15 to P28, PBS group as control. Scale bars: heart (top: 500 μm; bottom: 100 μm), liver (100 μm), spleen (200 μm), lung (500 μm), kidney (100 μm). **(D)** Representative immunofluorescence images and quantification showing the enrichment of DC499‑mFc (green) in the endothelial cells (red) in CCM lesions, n=3. Scale bar: 50 μm. Data are presented as mean ± SD. ** *P*<0.01, unpaired Student’s t-test for D.
